# The emerging role of Nrf2 in heart failure: From cardioprotection to therapeutic approaches

**DOI:** 10.1002/ehf2.15406

**Published:** 2025-09-08

**Authors:** Emiliano Fiori, Sergio Davinelli, Armando Ferrera, Alessandro Medoro, Carlo Barsali, Allegra Battistoni, Maurizio Volterrani, Massimo Volpe, Luciano Saso, Speranza Rubattu

**Affiliations:** ^1^ Cardiovascular Center AZORG Aalst Belgium; ^2^ Department of Clinical and Molecular Medicine Sapienza University Rome Italy; ^3^ Department of Medicine and Health Sciences “V. Tiberio” University of Molise Campobasso Italy; ^4^ Institute of Medicine and Sport Science National Italian Olympic Committee Rome Italy; ^5^ Cardiology Department Istituto di Ricovero e Cura a Carattere Scientifico (IRCCS) San Raffaele Roma Rome Italy; ^6^ Department of Physiology and Pharmacology "Vittorio Erspamer" Sapienza University Rome Italy; ^7^ IRCCS Neuromed Pozzilli IS Italy

**Keywords:** Nrf2, Heart failure, Oxidative stress, Chronic inflammation, Mitochondrial dysfunction, Cardioprotection, Cardiac rehabilitation, Cardio‐oncology

## Abstract

Heart failure (HF) is a multifactorial and pathophysiological complex syndrome, involving not only neurohormonal activation but also oxidative stress, chronic low‐grade inflammation, and metabolic derangements. Central to the cellular defence against oxidative damage is nuclear factor erythroid 2‐related factor 2 (Nrf2), a transcription factor that orchestrates antioxidant and cytoprotective responses. Preclinical in vitro and in vivo studies reveal that Nrf2 signalling is consistently impaired in HF, contributing to the progression of myocardial dysfunction. The loss of Nrf2 activity intersects a complex network of pathological processes involving neurohormonal activation, ischaemia–reperfusion injury, and sustained inflammation, exacerbating cardiac functional decline. Nrf2 deficiency diminishes resilience to clinical conditions such as hypertension, diabetic cardiomyopathy, and cancer therapy‐related cardiotoxicity, favouring the transition from initial cardiac dysfunction to overt HF. Initial evidence supports the therapeutic potential of Nrf2 modulation. Lifestyle interventions such as exercise training, various natural compounds, and established cardiovascular agents (e.g. sodium‐glucose cotransporter‐2 inhibitors) have been shown to restore Nrf2 activity. This review analyses the emerging role of Nrf2 as both a key player in HF pathogenesis and a promising therapeutic target, highlighting available evidence across HF phenotypes and addressing the controversies surrounding its pharmacological modulation.

## Graphical abstract

Nrf2 signalling is impaired in heart failure, contributing to oxidative stress, mitochondrial dysfunction, and ferroptosis, which drive neurohormonal activation, inflammation, and ischaemia‐reperfusion injury. This loss of Nrf2 activity exacerbates hypertension, diabetic cardiomyopathy, and cardiotoxicity, accelerating progression to overt heart failure. Therapeutic strategies that stimulate Nrf2, including exercise training, natural compounds, and guideline‐directed medical therapies (e.g. SGLT2 inhibitors), show promise in restoring redox balance, mitochondrial function, and myocardial resilience.

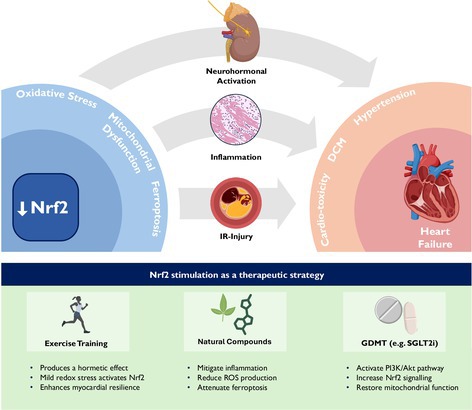

## Introduction

Despite advances in pharmacological and interventional treatments, heart failure (HF) remains a major contributor to morbidity and mortality worldwide, underscoring the need for novel therapeutic strategies. Traditionally, neurohormonal activation has been regarded as the central driver of HF progression. However, growing evidence suggests that oxidative stress, mitochondrial dysfunction, and chronic inflammation also affect the disease continuum, contributing to myocardial damage and further progression of the condition.^1^

Oxidative stress, defined as an imbalance between the excessive production of reactive oxygen species (ROS) and cellular antioxidant protective mechanisms, contributes to myocardial dysfunction and maladaptive cardiac remodelling. Among regulators of oxidative stress, nuclear factor erythroid 2‐related factor 2 (Nrf2) has emerged as a master transcription factor orchestrating cellular defence mechanisms against oxidative and inflammatory insults.^2^ Enhancing Nrf2 activity may serve as a promising therapeutic strategy to counteract HF progression.^3^

The pursue of this review is (i) to provide a comprehensive overview of the role of Nrf2 in HF pathogenesis; (ii) to explore the potential cardioprotective properties of Nrf2 in specific conditions such as hypertension, diabetic cardiomyopathy (DCM), and cancer therapy‐related cardiac dysfunction; (iii) to display the current research on Nrf2 modulation, including lifestyle modifications, natural compounds, and established guideline‐directed medical therapies (GDMT); and, finally, (iv) to provide a critical update on the clinical translation of Nrf2‐targeted strategies.

## Nrf2 pathway

### The role of Nrf2 in redox regulation

**Figure 1:**
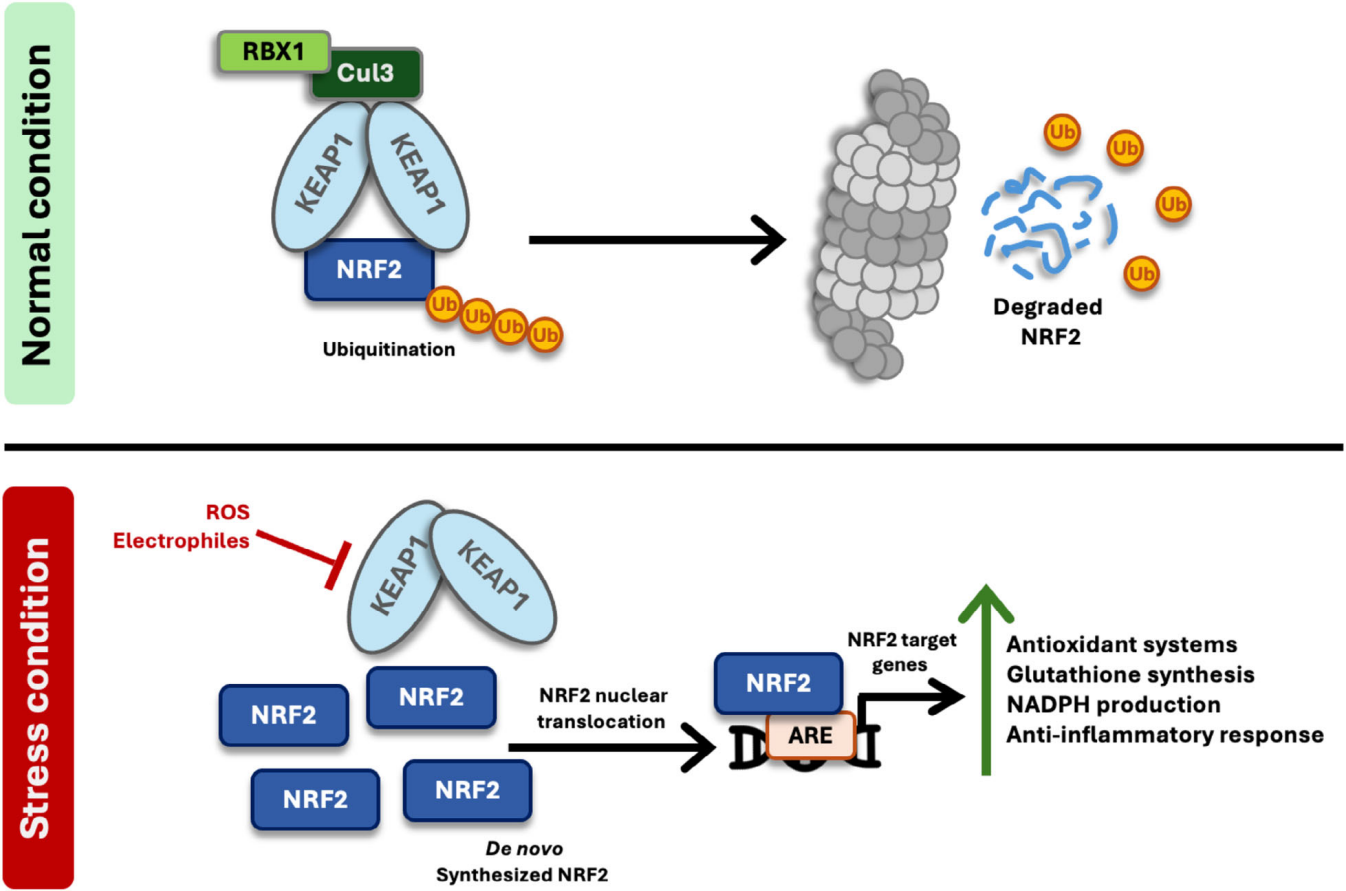
Nrf2 pathway. In homeostatic conditions, Nrf2 is tightly regulated by Kelch‐like ECH‐associated protein 1 (Keap1), which promotes its ubiquitination and degradation. In response to oxidative stress, Nrf2 dissociates from Keap1, translocates to the nucleus, and binds to antioxidant response elements (AREs), inducing the expression of cytoprotective genes involved in redox homeostasis, detoxification, mitochondrial function and anti‐inflammatory response. Cul3, Cullin 3; NADPH, nicotinamide adenine dinucleotide phosphate; RBX1, RING‐box protein 1; Ub, ubiquitin.

Nrf2 is a transcription factor belonging to the cap ‘n’ collar subfamily of basic region leucine zipper proteins. The regulatory network that controls Nrf2 activity links responses to oxidative stress, xenobiotic metabolism, and inflammatory signalling, maintaining cellular homeostasis and providing protection against environmental insults. Nrf2 function is mainly influenced by post‐translational modifications following its interaction with Kelch‐like ECH‐associated protein 1 (KEAP1), which serves as a redox sensor under normal conditions. In stable cells, KEAP1 binds to Nrf2, leading to ubiquitination and proteasomal degradation via the Cullin 3/Ring Box 1 E3 ubiquitin ligase complex (RBX1). During oxidative stress, modifications of critical cysteine residues on KEAP1 alter this interaction, allowing newly synthesized Nrf2 to move into the nucleus. There, Nrf2 interacts with small Maf proteins and binds to antioxidant response elements (ARE) in promoter or enhancer regions, activating transcription programmes that depend on cell type and the intensity and duration of environmental stress^4^, ^5^ (*Figure* *1*).

In addition to regulation by KEAP1, alternative pathways at the transcriptional, post‐transcriptional, and post‐translational levels, involving various proteins and epigenetic regulators, including microRNAs, further influence Nrf2 activity.^6^, ^7^

Multiple antioxidant enzymes are expressed under Nrf2 control, reducing oxidative damage by converting reactive molecules into less harmful compounds. Enzymes regulated by Nrf2 include superoxide dismutase (SOD), catalase (CAT), and haem oxygenase‐1 (HO‐1), each located in distinct subcellular compartments to support efficient neutralization of ROS and electrophiles. HO‐1 is activated in an oxidative environment and catalyses haem breakdown, generating bilirubin, a potent endogenous antioxidant.^8^, ^9^, ^10^ Nrf2 enhances cellular antioxidant defences through two related mechanisms that both target glutathione (GSH), a major intracellular antioxidant. First, Nrf2 increases the expression of glucose‐6‐phosphate dehydrogenase (G6PD), 6‐phosphogluconate dehydrogenase (6PGD), malic enzyme 1 (ME1), and isocitrate dehydrogenase 1 (IDH1), leading to higher NADPH levels. The resulting NADPH functions as a reducing agent, enabling the conversion of oxidized glutathione (GSSG) into GSH.^11^, ^12^ Second, Nrf2 plays a pivotal role in regulating the glutathione (GSH) system by controlling the expression of enzymes essential for its synthesis, including both the catalytic (GCLc) and modulatory (GCLm) subunits of glutamate‐cysteine ligase (GCL) and glutathione synthetase, as well as enzymes involved in GSH utilization and regeneration, such as glutathione reductase (GSR), glutathione peroxidase (GPX), and several glutathione S‐transferases (GSTs).^13^ Additionally, Nrf2 influences cystine uptake via the xCT transporter, which is crucial for GSH synthesis. While Nrf2 does not directly regulate NADPH production, it indirectly supports NADPH regeneration through its antioxidant response, thereby coordinating GSH activity and NADPH availability to maintain redox balance and enhance cellular defence against oxidative stress.^14^

### Nrf2 as a regulator of inflammatory response

Nrf2 modulates the pro‐inflammatory inflammatory signalling through both antioxidant‐dependent and redox‐independent mechanisms.^15^ A key anti‐inflammatory action of Nrf2 involves the suppression of nuclear factor kappa B (NF‐κB), a transcription factor regulating the expression of numerous pro‐inflammatory cytokine genes. Upon its activation, Nrf2 upregulates the expression of HO‐1, which exerts inhibitory effects on the IκB kinase (IKK) complex, a critical activator of NF‐κB signalling. HO‐1 activity prevents the phosphorylation and subsequent proteasomal degradation of IκBα, the inhibitory protein that sequesters NF‐κB in the cytoplasm under resting conditions. By stabilizing IκBα, HO‐1 effectively blocks NF‐κB nuclear translocation and its binding to DNA promoter regions.^16^, ^17^, ^18^ This inhibition of NF‐κB activation leads to a marked reduction in the transcription of downstream pro‐inflammatory mediators, including tumour necrosis factor‐alpha (TNF‐α), interleukin‐6 (IL‐6), and interleukin‐1 beta (IL‐1β). These cytokines are well‐established drivers of pathological myocardial remodelling, fibrosis, and contractile dysfunction in HF. Moreover, the suppression of NF‐κB signalling by Nrf2‐HO‐1 axis mitigates leukocyte infiltration and attenuates oxidative stress‐induced inflammatory amplification, thereby contributing to preserve cardiac tissue function.^19^ This mechanism highlights the crucial role of Nrf2 in modulating inflammatory pathways and highlights its potential as a promising therapeutic target in HF management. In addition to its effects through HO‐1, Nrf2 also reduces the expression of inflammatory genes through redox‐independent pathways. Indeed, Nrf2 can bind to promoter regions of key pro‐inflammatory cytokine genes independently of AREs. This non‐canonical binding facilitates the recruitment of histone deacetylases (HDACs) and other chromatin remodelling complexes, which promote the formation of a repressive chromatin environment characterized by histone deacetylation and reduced accessibility to transcriptional machinery. By altering the local chromatin state, Nrf2 inhibits the recruitment of RNA polymerase II to these gene loci, effectively suppressing transcriptional initiation and elongation of inflammatory mediators such as TNF‐α, IL‐6, and IL‐1β.^15^ The ability of Nrf2 to modulate gene expression through epigenetic and transcriptional repression highlights its function as a versatile regulator of inflammation, capable of fine‐tuning immune responses even when oxidative stress is minimal or absent. This redox‐independent anti‐inflammatory activity increases the therapeutic relevance of targeting Nrf2 in inflammatory cardiovascular diseases, including HF, in which dysregulated cytokine expression contributes to disease progression.^20^

Notably, Nrf2 suppresses NLRP3 inflammasome activation through several mechanisms. For example, Nrf2 promotes thioredoxin‐1 (Trx1) activity, which binds and inhibits thioredoxin interacting protein (TXNIP), thereby preventing TXNIP‐mediated NLRP3 inflammasome assembly, blocking caspase‐1 activation and subsequent IL‐1β maturation.^21^ Additionally, Nrf2 activation also induces HO‐1, whose anti‐inflammatory by‐products suppress mitochondrial ROS generation, a key activator of NLRP3, and inhibit NF‐κB signalling, which reduces pro‐IL‐1β synthesis^22^, ^23^ (*Figure* *1*). Given that cardiac fibroblasts exhibit higher NLRP3 expression, multi‐targeted inhibition via Nrf2 activation may reduce the ROS‐NLRP3‐IL‐1β amplification loop that drives myocardial remodelling in HF.

## Nrf2 network in the pathogenesis of cardiac dysfunction

Here we briefly review the evidence supporting the centrality of Nrf2 dysregulation in a network of pathological interactions leading to cardiac dysfunction.

### Mitochondrial dysfunction and ferroptosis

**Figure 2:**
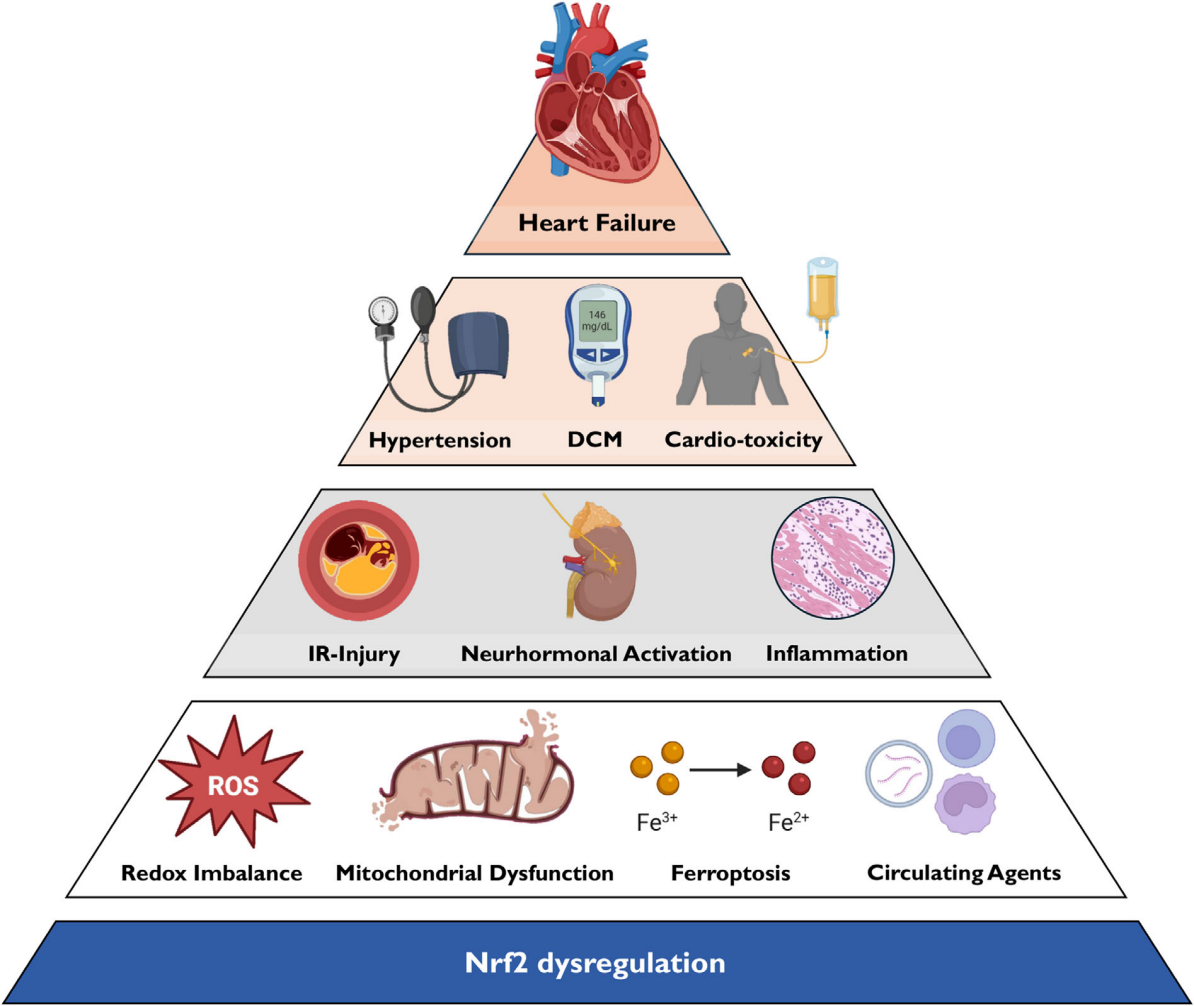
The role of Nrf2 in the network of HF pathogenesis. Nrf2 dysregulation reduces the cellular intrinsic defences against oxidative stress, inducing mitochondrial dysfunction and precipitating pathological responses like ferroptosis. This condition of redox imbalance is further amplified by circulating elements (exosomes vehiculating miRNA and peripheral blood mononuclear cells) that systemically propagate ROS. The breakdown of Nrf2 pathway is associated with a state of chronic inflammation, with the abnormal activation of neurohormonal systems and ultimately with a marked susceptibility to ischaemia/reperfusion injury. As a result, pre‐clinical studies support the cardioprotective role of Nrf2 in several conditions, such as hypertension, diabetes, and cardiotoxicity, where the inhibition of Nrf2 response has been associated with the progression to cardiac dysfunction and ultimately HF. DCM, diabetic cardiomyopathy; IR, ischaemia–reperfusion.

Mitochondrial dysfunction is a hallmark of HF, contributing to impaired energy metabolism and disease progression. It is often accompanied by oxidative stress, where excess ROS and insufficient antioxidant properties contribute to trigger cell death, inflammation, and fibrosis, accelerating cardiac decline^24^ (*Figure* *2*).

Nrf2 preserves the mitochondrial integrity by reducing oxidative stress. It regulates the expression of genes involved in mitochondrial dynamics, quality control, and membrane potential regulation. Specifically, Nrf2 activation boosts mitofusin 2 (Mfn2) expression while suppressing dynamin‐related protein 1 (Drp1), thereby favouring mitochondrial fusion over fission and enhancing mitochondrial stability.^25^ Furthermore, Nrf2 promotes mitophagy by upregulating PTEN‐induced kinase 1 (PINK1), ensuring the efficient clearance of dysfunctional mitochondria.^26^ This regulatory function of Nrf2 is vital for minimizing ROS accumulation and sustaining cellular energy balance.

Ferroptosis is a regulated form of cell death driven by iron accumulation, lipid peroxidation, and mitochondrial dysfunction. Nrf2 counteracts ferroptosis by modulating iron homeostasis, antioxidant defence, and lipid metabolism. It promotes the expression of ferritin heavy chain 1 (FTH1), which sequesters excess iron, thereby reducing the labile iron pool (LIP) and limiting iron‐driven oxidative stress. Additionally, Nrf2 enhances the activity of GPX4 and solute carrier family 7 member 11 (SLC7A11), key players in glutathione metabolism and lipid peroxide neutralization. Through these mechanisms, Nrf2 preserves redox balance, maintains mitochondrial integrity, and effectively suppresses ferroptotic cell death.^27^

### Circulating agents: Exosomes and leukocytes

Among extracellular vesicles (EVs), exosomes are specific small‐size (40–150 nm) organelles that originate from endosomes and are secreted by most cell types. These vesicles facilitate communication between cells: They carry proteins, microRNAs (miRNAs), lipids, and metabolites, which influence various cellular processes.^28^ In fact, EVs influence oxidative stress by regulating Nrf2 signalling in both the infarcted and failing heart.^29^

One of the mechanisms involves circulating miRNA‐1, which is significantly elevated in HF.^30^, ^31^ This miRNA contributes to cardiac oxidative stress by directly targeting SOD1, glutamate‐cysteine ligase catalytic subunit (Gclc), and glucose‐6‐phosphate dehydrogenase (G6PD), leading to increased ROS levels and heightened myocardial susceptibility to oxidative damage.^32^ Studies suggest that in response to TNF‐α stimulation, cardiac cells selectively incorporate miRNA‐1 into EVs, which are then secreted to impair Nrf2 translation in recipient cells, disrupting their oxidative stress defences.^33^ Conversely, miRNA‐200a, another EV component, has been shown to epigenetically inhibit Keap1 expression, thereby activating Nrf2/ARE signalling and reducing oxidative stress, particularly in the context of hepatic fibrosis.^34^ However, in the setting of ageing and myocardial infarction (MI), EVs from aged mesenchymal stem cells (MSC‐EVs) exhibit a downregulation of miRNA‐200a, leading to the restoration of Keap1 expression. This results in a significant weakening of Nrf2 signalling and a loss of the cardio‐protective effects typically conferred by young MSC‐EVs post‐MI.^35^

In addition, HF is characterized by altered mitochondrial function and structure in circulating leucocytes,^36^ this arising from a reduced ROS clearance in the lungs and peripheral tissues combined with myocardial ROS generation. Consequently, peripheral blood mononuclear cells (PBMCs) can amplify cardiovascular oxidative stress, delivering ROS to endothelial cells and back to the myocytes, creating a vicious loop of oxidative stress propagation. Nrf2 dysregulation could potentially catalyse this chain reaction^37^ by reducing the antioxidant defence of both myocardial and circulating cells.^38^

This suggests that circulating leucocytes and vesicles partially mediate the intercellular and inter‐organ communication, influencing inflammation, oxidative balance, and cardiac remodelling.^29^

### Neurohormonal dysregulation

The central nervous system (CNS) participates in the regulation of the cardiovascular system by modulating heart rate and blood pressure through the autonomic nervous system (ANS). In view of the high sensitivity of neurons to oxidative stress, it is essential to maintain redox balance for an optimal function of both the CNS and the cardiovascular physiology. However, in chronic HF and hypertension, Nrf2 downregulation in central autonomic neurons leads to increased oxidative stress and sympathetic drive, thus contributing to autonomic dysregulation and worsening of these CV conditions^39^ (*Figure* *2*).

Studies have shown that animal models of HF and hypertension exhibit both reduced production of antioxidant enzymes and decreased expression of Nrf2 in the CNS.^40^ The ANS, in turn, contributes to overproduction of ROS via pro‐oxidant signalling such as norepinephrine, angiotensin II (Ang II), and cytokines. In the CNS, neuronal ROS modulate ion channels activity and enhance the sensitivity of pre‐sympathetic neurons.^3^ This enhanced sensitivity leads to upregulation of the renin–angiotensin–aldosterone system (RAAS), primarily affecting the CV system through the actions of Ang II, which in turn activates NADPH oxidase enzymes and increases ROS production.^39^

In summary, Nrf2 downregulation promotes oxidative stress, which amplifies sympathetic drive and worsens CV dysfunction.

### Latent inflammation

The Nrf2 dysregulation leads to latent inflammation by enhancing pro‐inflammatory signalling pathways and increasing the innate immune response (see Nrf2 as a regulator of inflammatory response section).

Nrf2 modulates macrophage activity, recruitment, activity and also interleukin release.^37^ In mice models, Nrf2 has been shown to regulate the expression of type I interferon receptor (IFNAR), favouring the development of pro‐resolving non‐classic macrophages (NCMs, or M2) over pro‐inflammatory classic macrophages (CMs, or M1), thus contributing to the resolution of chronic inflammation.^37^ In the study conducted by Chen *et al*.,^41^ Nrf2 knockout mice demonstrate a role of agonist‐induced activation of IL‐6/STAT3 signalling pathway, which leads to cardiac hypertrophy, fibrosis, oxidative stress, and inflammation.

The disruption of Nrf2 signalling contributes to the development of a low‐grade inflammatory state, which may worsen cardiac function leading to development of hypertrophy and fibrosis, which are key common features in the pathophysiology of heart failure with preserved ejection fraction (HFpEF).^42^

### Ischaemia–reperfusion injury

The ischaemia–reperfusion injury (IRI) occurs when blood perfuses an ischaemic area, damaging the area itself.^43^, ^44^ It is mediated by oxidative stress, calcium overload, and mitochondrial dysfunction, all leading to cellular death,^45^ aggravating the initial ischaemic insult and hampering the potential benefit of blood flow restoration (e.g. myocardial recovery).

HO‐1, NADPH‐quinone oxidoreductase 1 (NQO1), and GPX2, which are produced upon binding of NrF2 to ARE, contribute to a reduction of ROS and protect the myocardium from reperfusion injury.^46^ In fact, in Langendorff‐perfused hearts with IRI, the administration of 4‐hydroxy‐2‐nonenal, which activates Nrf2, increases the production of glutathione,^47^, ^48^ reduces ROS, and downregulates inflammation. These effects translate in left ventricular function improvement.

Nrf2 also contributes to recruitment and activation of monocytes/macrophages and granulocytes in myocardial tissue with IRI,^37^ facilitating the removal of damaged mitochondria, decreasing apoptosis, and helping to remove the collagen from the myocardial tissue itself.^49^ Furthermore, the activation of Nrf2 contributes to reducing the size of infarcted areas in the heart, decreasing vascular damage, and preventing the accumulation of neutrophils, which would otherwise lead to fibrosis and cardiac remodelling.^50^

## Nrf2 and cardioprotection

### Hypertension and cardiac remodelling

Prolonged elevated blood pressure induces cardiac mechanical and oxidative stress, leading to structural changes such as myocardial hypertrophy, fibrosis, and ultimately HF.^51^ These changes are mediated by various cellular and molecular mechanisms, including the activation of oxidative stress, pro‐inflammatory pathways, and alterations in myocardial metabolism.^52^

In normal conditions, Nrf2 activation helps to maintain redox balance and could be involved in in protecting the heart from hypertension‐mediated organ damage.^53^ In the context of hypertension, Nrf2 activation can reduce myocardial oxidative stress and mitigate the activation of pro‐inflammatory cytokines, attenuating the progression of fibrosis and hypertrophy.^54^ In animal models of increased cardiac afterload, activation of Nrf2^55^ and its downstream genes HO‐1^56^ and SOD2^57^ provide a novel mechanism to protect the heart against pressure overload‐induced pathological remodelling and HF via suppressing oxidative stress.

Several preclinical studies have demonstrated that pharmacological activation of Nrf2 can protect against hypertension‐induced cardiac remodelling.^58^ Nrf2 activation has been shown to improve left ventricular function, reduce myocardial fibrosis, and attenuate the hypertrophic response in experimental models of hypertension.^59^ Moreover, Gao *et al*.^60^ indicated that Nrf2 gene deletion in mice elevates blood pressure, increases sympathetic outflow, and impairs baroreflex function potentially by impaired antioxidant enzyme expression.

These findings suggest that Nrf2 activation may represent a promising therapeutic strategy for preventing or reversing cardiac remodelling associated with hypertension, although clinical evidence has not been produced.

### Diabetic cardiomyopathy

DCM is characterized by metabolic dysregulation, oxidative stress, and inflammation, leading to myocardial fibrosis and dysfunction.^61^ While Nrf2 is adaptively overexpressed to reduce diabetic damages in the early stages of the disease, in later stages, cardiac antioxidant function is severely impaired and characterized by a decrease in cardiac Nrf2 expression.^62^

Nrf2 may protect the diabetic heart by modulating glucose and lipid metabolism, reducing inflammation, and strengthening antioxidant defences.^63^ Preclinical studies point out how myocardial susceptibility to hyperglycaemia is exaggerated in the absence of Nrf2 and its downstream pathway.^64^ Conversely, the activation of Nrf2 and its downstream effectors has been shown to reduce myocardial fibrosis and improve left ventricular function in animal models of DCM.^65^, ^66^, ^67^

Mechanistically, the cardioprotective effects of Nrf2 depend on its antioxidant activity but also partially through improving glucose and lipid metabolism.^68^ Specifically, Xu *et al*.^65^ demonstrated in mice that asiaticoside, a triterpenoid saponin extracted from *Centella asiatica* , can alleviate DCM myocardial injury by improving mitochondrial status, enhancing autophagy, and reducing oxidative stress through AMPK/Nrf2 pathway. Nrf2‐mediated regulation of autophagy contributes to cellular homeostasis and mitigates glucotoxicity and lipotoxicity in diabetic hearts.^69^

The excessive oxidative environment coupled with hampered ROS scavenging systems and iron accumulation^70^ make the myocardium of diabetic patients particularly vulnerable to ferroptosis.^71^ Nrf2 could counteract the myocardial cell loss at least in part by defending them from the catastrophic chain reaction of ROS imbalance, lipid peroxidation, mitochondrial dysfunction, and ferroptosis.^72^ Nuclear translocation of Nrf2 induced by curcumin increases the expression of oxidative scavenging factors (such as HO‐1), reduces excessive GPx loss, and inhibits glucose‐induced ferroptosis in cardiomyocytes.^73^

The multiple effects of Nrf2 potentially protecting cardiomyocytes from diabetes‐related damage support its potential as a therapeutic target for preventing diabetic cardiomyopathy, although this has yet to be confirmed in human studies.

### Cancer therapy‐related cardiac dysfunction

The advancement of oncological therapies has greatly improved cancer prognosis but also highlighted the issue of treatment‐related cardiotoxicity. Anthracyclines, commonly used in solid and haematological cancers, are topoisomerase II inhibitors with well‐documented CV adverse effects. Their most common dose‐dependent toxicity is left ventricular dysfunction, which can progress to overt HF. Although not fully understood, several key molecular mechanisms have been implicated in this cardiotoxicity. Anthracyclines exert direct effects on DNA and protein synthesis, disrupt calcium metabolism, and promote myofibre degeneration.^74^

Nrf2 has been implicated in the pathogenesis of doxorubicin (DOX)‐induced cardiotoxicity. Preclinical data link DOX administration to oxidative stress, impaired autophagy, and the accumulation of polyubiquitinated protein aggregates. These effects are markedly exacerbated in Nrf2 knockout (Nrf2−/−) mice. Conversely, in cultured cardiomyocytes, Nrf2 overexpression has been shown to alleviate DOX‐induced autophagy impairment and protein aggregation while also reducing DOX‐induced cytotoxicity.^75^

Moreover, the activation of Nrf2 protects cardiomyocytes by inhibiting ROS production and, consequently, downregulating the mitochondrial apoptotic pathway in DOX‐treated cardiomyocytes.^76^ There is also evidence that DOX increases oxidative stress and inflammation by suppressing NRF2 expression, potentially through interference with the protein kinase B (AKT) and the SIRT1/LKB1/AMPK/Nrf2 signalling pathways.^77^

Interestingly, following DOX treatment, a time‐dependent increase in myocardial Nrf2 expression (at both mRNA and protein levels), as well as in downstream targets such as HO‐1 and NQO‐1, has been observed.^75^ It has been proposed that DOX may decrease Keap1 expression in a redox‐sensitive manner, leading to a modest upregulation of Nrf2‐typically insufficient to counteract the extensive oxidative damage in the heart.^78^

Collectively, these findings suggest that Nrf2 acts as an endogenous suppressor of DOX‐induced cardiotoxicity, primarily by modulating oxidative stress, inflammation, and autophagy in cardiac tissue. Thus, Nrf2 emerges as a promising therapeutic target for managing DOX‐related cardiac dysfunction, either directly or via regulation of Keap1.^79^ In this regard, the activation of Nrf2 by miR‐152 has shown potential in mitigating DOX‐induced cardiotoxicity by reducing oxidative stress, inflammation, and apoptosis. Additionally, numerous natural compounds—including phytochemicals—have demonstrated the ability to activate Nrf2, offering a promising strategy to attenuate DOX‐induced cardiotoxic effects.^77^

Taken together, this evidence identifies Nrf2 as a precious ally in the defence of the cardiovascular system from a wide range of different insults. In the framework of HF, Nrf2 assumes a central position in a network of pathological processes and clinical conditions, acting as the pivotal gear of a new ‘oxidative stress’ paradigm^80^ in the progression of the syndrome (*Figure* *2*). Modulation of Nrf2 to prevent HF or slow its progression is, therefore, an appealing therapeutic strategy.

## Nrf2 modulation as target of therapy

### Exercise‐induced modulation of Nrf2

Regular physical activity is a well‐established strategy for cardiovascular health, and Nrf2 appears to mediate part of its benefits.^81^ Exercise‐induced shear stress and mild oxidative stress activate Nrf2 signalling, leading to upregulation of antioxidant enzymes such as SOD and GPX.^82^ This adaptive response enhances myocardial resilience against ischaemia–reperfusion injury, improves endothelial function, and reduces chronic inflammation.^83^ Merry and Ristow^84^ displayed that following exercise training mice with impaired Nrf2 expression have reduced exercise performance, energy expenditure, mitochondrial volume, and antioxidant activity. Furthermore, exercise training has been shown to restore Nrf2 activity in aged and diseased hearts, suggesting its role in maintaining redox homeostasis and promoting longevity.^85^ Done *et al*.^86^ showed that a single session of submaximal aerobic exercise is sufficient to activate Nrf2 at the whole cell level in both young and older adults but that nuclear import is impaired with aging.

The exercise‐induced Nrf2 activation demonstrates a hormetic effect, where repeated exposure to moderate oxidative challenges enhances the cell's adaptive capacity against subsequent stressors. Regular endurance exercise has been shown to increase basal Nrf2 levels and enhance nuclear accumulation following acute bouts of exercise, particularly in skeletal muscle and cardiac tissue, suggesting tissue‐specific adaptations.^87^ Intensity and duration of exercise critically determine the Nrf2 response. Moderate‐intensity exercise consistently activates the Nrf2 pathway, while both high‐intensity and prolonged exhaustive exercise may overwhelm this protective mechanism, highlighting the importance of appropriate exercise prescription when targeting Nrf2 as a therapeutic strategy.^88^, ^89^

The Nrf2‐mediated benefits of exercise extend beyond antioxidant protection to include anti‐inflammatory effects, metabolic regulation, and mitochondrial biogenesis. These pleiotropic effects position exercise‐induced Nrf2 modulation as a promising therapeutic target for conditions characterized by oxidative stress and inflammation, including HF.^90^

### Modulation of Nrf2 by natural compounds

Numerous experimental findings indicate that several bioactive food compounds, particularly polyphenols and sulfur‐rich compounds, may exert cardioprotective effects by modulating the activity of Nrf2.

#### Polyphenols

Polyphenols, a heterogeneous group of naturally occurring compounds characterized by one or more phenolic rings, exert cardioprotective effects through an interconnected network of mechanisms involving modulation of integrin signalling and activation of the Nrf2. Integrins, which regulate cell adhesion, migration, and survival, are indirectly influenced by polyphenols through reduced expression of adhesion molecules such as intercellular adhesion molecule‐1 (ICAM‐1) and decreased platelet activation. Likewise, polyphenols activate Nrf2 cascade by disrupting Keap1. This response is further enhanced by integrin‐initiated signalling cascades, including phosphoinositide 3‐kinase/protein kinase B (PI3K‐AKT)‐mediated phosphorylation of Nrf2.^91^, ^92^, ^93^

Resveratrol is a polyphenol well known for its antioxidant and anti‐inflammatory properties, which contribute to its potential in HF management. Preclinical studies demonstrate that administration prior to HF onset prevents cardiac hypertrophy and preserves myocardial function through SIRT1‐dependent AMPK activation.^94^ Resveratrol also mitigates diabetic cardiomyopathy, enhances cardiomyocyte survival, and improves cardiac function by upregulating Nrf2 and antioxidant enzymes (e.g. SOD2) while restoring sarcoplasmic reticulum calcium ATPase activity.^95^, ^96^, ^97^ Likewise, quercetin, a flavonoid belonging to the flavonol subclass of polyphenols, has been shown to exert beneficial effects in cultured cardiomyocytes exposed to cisplatin by inducing HO‐1 expression, increasing SOD levels, maintaining mitochondrial function, and reducing oxidative stress.^98^ Kaempferol is another flavonoid with cardioprotective effects. It activates the Nrf2‐ARE pathway and suppresses pro‐inflammatory cytokines such as TNF‐α and IL‐6 by inhibiting NF‐κB signalling, reducing endothelial dysfunction and vascular inflammation. Kaempferol also protects against Ang II‐induced cardiac remodelling and oxidative vascular injury. Although its poor solubility and low bioavailability limit clinical use, advanced delivery systems are being explored to enhance its therapeutic potential.^99^, ^100^, ^101^

Ferroptosis contributes to cardiomyocyte loss in HF. Curcumin, a turmeric‐derived polyphenol, shows cardioprotective effects via Nrf2 activation. In diabetic and high‐glucose‐treated cardiomyocytes, it activates the Nrf2‐ARE pathway, improves myocardial structure; reduces fibrosis, ROS, and lipid peroxidation; and restores GPX4 while suppressing Cox1 and Acsl4. These actions, via the Nrf2/HO‐1/GPX4 axis, reduce oxidative stress and ferroptosis.^73^ Among isoflavones, calycosin reduces doxorubicin‐induced ferroptosis by modulating Nrf2, SLC7A11, and GPX4 in a rat model of HF.^102^ A randomized clinical trial in ischaemic stroke patients showed that soybean isoflavones enhance Nrf2 and SOD expression, lowering 8‐isoprostane (8‐iso‐PGF2α), malondialdehyde (MDA), IL‐6, and tumour necrosis factor‐alpha (TNF‐α).^103^

Chalcones, flavonoids with cardioprotective activity, modulate targets like angiotensin‐converting enzyme (ACE), potassium and calcium channels, and cyclooxygenase enzymes (COX‐1)‐1 and affect lipid metabolism via lipoprotein lipase (LPL) and cholesteryl ester transfer protein (CETP) inhibition.^104^ Cardamonin protects against septic and diabetic cardiomyopathy by improving glucose metabolism, reducing injury, and inhibiting inflammation via Nrf2‐ARE activation and NF‐κB inhibition. KEAP1 is a key target.^105^, ^106^ Butein, another chalcone, improves cardiac function in HF rats by enhancing antioxidant defences via ERK/Nrf2 signalling and reducing oxidative damage.^107^

#### Sulfur‐rich compounds

In addition to polyphenols, sulfur‐rich compounds such as sulforaphane and allicin have gained attention for their cardioprotective effects in the context of HF. These food‐derived molecules exert many of their antioxidant and anti‐inflammatory effects through activation of the Nrf2 signalling pathway.

In an experimental model of diabetic cardiomyopathy, sulforaphane, a well‐characterized isothiocyanate found in cruciferous vegetables, has been reported to prevent ferroptosis‐driven HF by activating Nrf2 and AMPK pathways. Moreover, the cardioprotective effect of sulforaphane has been shown to upregulate the expression of ferritin and SLC7A11, key proteins that counteract ferroptosis, which in turn protects cardiac tissue from damage induced by advanced glycation end‐products (AGEs).^108^ Several studies have also demonstrated that sulforaphane may prevent Ang II‐induced myocardial and aortic injury by promoting Nrf2 nuclear translocation through epigenetic modifications, including reduced DNA methylation and increased histone acetylation at the Nrf2 promoter, along with inhibition of histone deacetylases (HDACs) and DNA methyltransferases (DNMTs). Additionally, activation of Nrf2‐mediated antioxidant defences by sulforaphane is linked to the ERK/GSK‐3β/Fyn signalling pathway, further highlighting that the prevention of Ang II‐induced oxidative stress and HF by sulforaphane is mediated by Nrf2 through multiple molecular mechanisms.^109^, ^110^, ^111^

The garlic‐derived sulfur compound allicin also exerts cardioprotective effects through activation of the Nrf2 pathway, targeting key mechanisms involved in HF. In an experimental model of septic cardiomyopathy, allicin prevented LPS‐induced myocardial injury by reducing oxidative stress and proinflammatory cytokine production, activating the Nrf2/HO‐1 axis, and suppressing NLRP3 inflammasome signalling.^23^ It has also been shown that allicin exerts protective effects against Ang II‐induced cardiac hypertrophy by enhancing the expression of Nrf2‐regulated antioxidant proteins, including NQO1 and GPX, while reducing the accumulation of ROS and protein carbonyls.^112^

### Guideline‐directed medical therapy

After reviewing the effects of natural Nrf2 modulators in various in vitro and in vivo models of cardiac injury, we shift our focus to the impact of established cardiovascular drugs on Nrf2 (*Table* 1).

**Table 1:** Effects of guideline‐directed medical therapies on NRF2 pathway: summary of both in vitro and in vivo studies.

Molecule	Dosage	Experimental model	Experimental design	Effect	Reference (*N*)	Clinical indication
Sodium‐glucose cotransporter‐2 inhibitors (SGLT2i)						
Dapagliflozin	20 μM for 24 h	In vitro: rat myoblast cell (H9c2) cultured with 10 μM doxorubicin for 24 h (DOX)	Three groups: H9c2 DOX exposed to DAPA (DOX + DAPA) compared with H9c2 (DOX) and naive H9c2 as reference control (CN)	Restores PI3K/Akt signalling **Increases Nrf2, NQO‐1 and SOD2, gene expression** Mitigates p38/NF‐κB inflammatory signalling pathway Reduces ROS production Improves mitochondrial function	Hsieh *et al*.^113^	**HFrEF** **HFmrEF** **HFpEF** **CKD** **Diabetes**
	0.1 mg/kg/day	In vivo: Doxorubicin‐induced cardiomyopathy (DIC) model (male Sprague–Dawley rats, 12 mg/kg doxorubicin cumulative dose)	Two groups: DIC model exposed to DAPA (DIC + DAPA) compared with DIC as control (CN)	Reduces markers of both hypertrophy (ANP and BNP) and fibrosis (phospho‐Smad3, collagen I, fibronectin, and α‐SMA) Improves cardiac remodelling and LVEF		
Dapagliflozin	1 mg/kg/day per 10 weeks	In vivo: HF model (rabbit with ascending aorta circumferential ligation)	Three groups: HF model exposed to DAPA (HF + DAPA) compared to HF model (HF) and sham procedure as reference control (CN)	**Increases Nrf2, HO‐1, and GPX4 gene expression** Increases serum GSH‐Px and SOD activity Decreases IL‐1β, IL‐6, and TNF‐α levels Decreases cardiac iron levels Improves cardiomyocyte hypertrophy, degeneration and necrosis Improves cardiac remodelling and LVEF	Zhang *et al*.^114^	
Empagliflozin	10 mg/kg/day per 20 weeks	In vivo: Diabetic cardiomyopathy (DCM) model (BKS.Cg‐Dock7 m +/+ Lepr db mice)	Three groups: DCM model exposed to EMPA (DCM + EMPA) compared to DCM and wild‐type mice (littermate C57BLKS/J) as reference control (CN)	**Increases Nrf2, NQO‐1, and SOD2 genes expression** Reduces ROS production Improves mitochondrial structure, function, and dynamic Improves cardiac remodelling and LVEF	Wang *et al*.^115^	**HFrEF** **HFmrEF** **HFpEF** **CKD** **Diabetes**
Canagliflozin	10 μM for 12 h	In vitro: HL‐1 cardiomyocyte treated with 1 μM isoprenaline for 12 h	Three groups: HL‐1 cardiomyocyte treated with canagliflozin (CANA), HL‐1 treated with isoprenaline (ISO) and HL‐1 co‐treated with ISO and canagliflozin (ISO + CANA)	Restores PI3K/Akt signalling Activates AMPK **Increases Nrf2, HO‐1, SOD2 and GPX4 genes expression** Reduces ROS production	Hasan *et al*.^116^	**Diabetes**
	5 mg/kg/day per 1 week	In vivo: Tachi‐induced cardiomyopathy (TIC) model (subcutaneous injection of isoprenaline at 50 mg/kg twice a week)	Four groups: TIC model (ISO), control group exposed to canagliflozin (CANA) TIC model subsequently treated with canagliflozin (ISO + CANA) and naïve control (CN) as reference	Attenuates myocardial apoptosis and fibrosis Reduces plasmatic markers of myocardial injury (CK‐MB)		
Soluble guanylate cyclase (sGC) stimulator						
Vericiguat	1 mg/kg/day per 8 weeks	In vivo: DIC model (male Sprague Dawley rats, 12 mg/kg doxorubicin cumulative dose)	Three groups: DCM model exposed to Vericiguat (DIC + Ver) compared to DCM and normal rats as reference control (CN)	Upregulation NO‐cGMP‐PKG signalling **Increases Nrf2 gene expression** Restores plasma SOD levels Attenuates myocardial apoptosis and fibrosis Reduces plasma NT‐proBNP levels Improves cardiac remodelling and LVEF	Chen *et al*.^119^	**Worsening HFrEF**
Beta‐blockers						
Carvedilol	0.25 and 2.5 μM for 3 h	In vitro: Rat myoblast cell (H9c2) and human induced pluripotent stem cell‐derived cardiomyocyte (iPSC‐CM) cultured with 1 μM doxorubicin for 24 h (DOX)	Two groups: H9c2 and iPSC‐CMs pretreated with carvedilol and then cultured with doxorubicin (CV + DOX) compared to non‐pretreated cells as reference control (DOX)	**Maintains Nrf2, NQO‐1, and SOD2 gene expression** Reduces ROS production Preserves mitochondrial function Attenuates apoptosis signalling	Uche *et al*.^122^	**HFrEF** **HFmrEF** **Chronic coronary syndrome**
Bisoprolol (+ trimetazidine)	8 and 60 mg/kg/day per 3 weeks	In vivo: Arsenic trioxide (ATO)‐induced myocardial injury (male Wistar rats, 7.5 mg/kg per 2 weeks)	Five groups: Normal control group (normal saline), ATO group, pretreatment with BIS + ATO, pretreatment with TMZ + ATO, and pretreatment combination group (BIS + TMZ + ATO)	Activate PI3K/Akt signalling pathway **Increase Nrf2 gene expression** Reduce ROS production Increase myocardial GSH‐Px and SOD activity Reduce myocardial inflammation Reduce myocardial necrosis, apoptosis and fibrosis Reduce plasmatic myocardial injury markers (LDH, CK‐MB, c‐Tn)	Ahmed *et al*.^124^	**HFrEF** **HFmrEF** **Chronic coronary syndrome**
Statins						
Pravastatin	2 mg/kg/day per 1 week	In vivo: ischaemia/reperfusion (I/R) model (30 minutes LAD coronary artery ligation)	Three groups: pretreatment with pravastatin (I/R + P), I/R without pretreatment and sham procedure as reference control (CN)	Inhibits miR‐93 expression **Increases Nrf2, NQO‐1, and SOD2 gene expression** Increases myocardial GSH‐Px and SOD activity Attenuates apoptosis signalling Reduces plasmatic myocardial injury markers (LDH, CK‐MB, c‐Tn) Reduces myocardial infarction area	Liu *et al*.^132^	**Hypercolesterolaemia**
Rosuvastatin	10 μM for 6 h	In vitro: Rapid pacing of cultured atrial‐derived myocytes (HL‐1 cardiomyocytes)	Two groups: HL‐1 pretreated with rosuvastatin and then subjected to field stimulation compared to non‐pretreated cells as reference control (CN)	Activates PI3K/Akt signalling pathway **Increases Nrf2 and HO‐1 genes expression** Reduces ROS production Attenuates structural and electrical remodelling	Yeh *et al*.^133^	**Hypercolesterolaemia**
	20 mg/kg/day per 1 week	In vivo: Tachi‐induced cardiomyopathy (TIC) model (male Wistar rats, RA burst pacing at 15 Hz)	Two groups: TIC model pretreated with rosuvastatin (TIC + RO) compared to non‐pretreated rats as reference control	Activates PI3K/Akt signalling pathway **Increases Nrf2 and HO‐1 gene expression** Reduces ROS production		
Other lipid‐lowering agents (LLAs)						
Omega‐3 polyunsaturated fatty acids (PUFAs)	100 μM for 48 h	In vitro: Human aortic endothelial cells (HAECs) incubated H_2_O_2_ (100 μM) for 15 min	Two groups: HAECs pretreated with PUFAs and then incubated with H_2_O_2_ compared to not‐pretreated cells as reference control (CN)	Attenuate DNA damage **Increase Nrf2 and SOD2 genes expression** Reduce ROS production Attenuate cell senescence	Sakai *et al*.^128^	**HFrEF** **Hypertrigliceridaemia**
Ezetimibe	1.5 mg/kg/day per 4 weeks	In vivo: DIC model (Balb/c mice of both sexes, 16 mg/kg doxorubicin cumulative dose)	Four groups: normal control (NC), NC group treated with ezetimibe (NC + E), DIC control and DIC treated with ezetimibe (DIC + E)	Reduces plasmatic markers of myocardial injury (CK‐MB, LDH) Improves cardiac atrophic index and heart weight‐to‐body weight ratio	Vashi *et al*.^131^	**Hypercolesterolaemia**

Akt, protein kinase B; AMPK, AMP‐activated protein kinase; ANP, atrial natriuretic peptide; ATO, arsenic trioxide; BIS, bisoprolol; BNP, brain‐derived natriuretic peptide; CANA, canagliflozin; cGMP, cyclic guanosine monophosphate; CK‐MB, creatine kinase MB; cTn, troponin C; DAPA, dapagliflozin; DCM, diabetic cardiomyopathy; DOX, doxorubicin; EMPA, empagliflozin; GPX4, glutathione peroxidase 4; H_2_O_2_, hydrogen peroxide; HAECs, human aortic endothelial cells; HO‐1, haem oxygenase‐1; I/R, ischaemia–reperfusion; IL‐1β, interleukin 1 beta; IL‐6, interleukin 6; iPSC‐CMs, human induced pluripotent stem cell‐derived cardiomyocyte; ISO, isoprenaline; LAD, left anterior descending coronary artery; LDH, lactate dehydrogenase; LVEF, left ventricular ejection fraction; NF‐κB, nuclear factor kappa‐light‐chain‐enhancer of activated B cells; NO, nitric oxide; NQO‐1, NAD(P)H dehydrogenase (quinone 1); Nrf2, nuclear factor erythroid 2‐related factor 2; NT‐proBNP, N‐terminal pro‐BNP; PI3K, phosphatidylinositol 3‐kinase; PKG, protein kinase G; PUFAs, poly‐insatured fatty acids; RA, right atrium; RO, rosuvastatin; ROS, reactive oxygen species; SOD, superoxide dismutase; TIC, tachi‐induced cardiomyopathy; TMZ, trimetazidine; TNF‐α, tumour necrosis factor alfa; Ver, vericiguat.

#### Sodium‐glucose cotransporter‐2 inhibitors

Within the spectrum of HF medications, sodium‐glucose cotransporter‐2 (SGLT2) inhibitors exhibit a class‐wide effect in stimulating the Nrf2‐HO1 pathway. Several preclinical studies demonstrated how dapagliflozin reduces ROS production and enhances the antioxidant defence system (SOD2, GPx4) in both in vitro and in vivo models of DOX‐induced cardiotoxicity and HF.^113^, ^114^, ^115^

Additionally, dapagliflozin mitigates inflammatory signalling,^113^ improves mitochondrial function, and reduces myocardial hypertrophy and fibrosis. These effects contribute to positive cardiac remodelling and recovery of left ventricular ejection fraction (LVEF). Similar cardioprotective effects were observed with empagliflozin in an animal model of DCM^116^ and with canagliflozin in a tachycardia‐induced cardiomyopathy (TIC) model.^117^

SGLT2 inhibitors modulate Nrf2 signalling through two primary mechanisms: activation of the PI3K/Akt pathway, which promotes Nrf2 nuclear translocation,^118^ and the activation of AMPK, which phosphorylates and inhibits GSK3β, preventing Nrf2 degradation.^119^

#### Soluble guanylate cyclase stimulator

Vericiguat is the first soluble guanylate cyclase (sGC) stimulator approved for the management of worsening HF. Its impact on Nrf2 has been investigated in an in vivo model of DOX‐induced cardiotoxicity.^120^ In rats exposed to DOX, vericiguat increased Nrf2 expression, restored SOD levels, and attenuated myocardial apoptosis. Following treatment, vericiguat was associated with reduced NT‐proBNP levels and improved cardiac function. These findings support the hypothesis that NO‐cGMP‐PKG signalling plays a direct role in modulating the Nrf2 pathway.^121^

#### Beta‐blockers

Evidence on the interaction between beta‐blockers and Nrf2 remains limited. Among them, carvedilol, a non‐selective beta‐blocker with intrinsic antioxidant properties,^122^ appears to exert a specific cardioprotective effect mediated by Nrf2.^123^ In this in vitro study on cardiomyocytes exposed to toxic levels of DOX, Uche *et al*. demonstrated that pretreatment with carvedilol reduces ROS production, mitigates mitochondrial dysfunction, and attenuates apoptosis signalling while preserving Nrf2 gene expression. The effects of carvedilol on the Nrf2/ARE pathway have also been well documented in neuronal cells,^124^ where the beta‐blocker has shown protective effects against oxidative stress toxicity.

Similarly, pretreatment with bisoprolol, a beta‐1 selective blocker, was found to increase Nrf2 gene expression via the PI3K/Akt pathway in an animal model of myocardial injury.^125^ This upregulation resulted in reduced ROS production, myocardial inflammation, apoptosis, and fibrosis in response to arsenic trioxide exposure.

The anti‐inflammatory and oxidative defence mechanisms of beta‐blockers may be exerted either directly on myocardial tissue or through an off‐target immune modulation of circulating monocytes, potentially via the Nrf2/ARE system.^126^

#### Lipid‐lowering agents

According to US guidelines on HF management, omega‐3 polyunsaturated fatty acids (PUFAs) may be considered as an adjunctive therapy to reduce mortality and cardiovascular hospitalizations in HF patients.^127^ PUFAs exhibit pleiotropic antioxidant properties, which may contribute to their beneficial effects in HF.^128^

Notably, Sakai *et al*. demonstrated that a combination of eicosapentaenoic acid (EPA) and docosahexaenoic acid (DHA) protects endothelial cells from ROS‐induced DNA damage and senescence by upregulating Nrf2 and SOD2 gene expression.^129^ Endothelial dysfunction is responsible for atherosclerosis development but also chronic vasoconstriction and microvascular dysfunction,^130^ two conditions tightly associated with the progression of HF.

Ezetimibe is a selective inhibitor of cholesterol absorption, extensively employed for managing hypercholesterolaemia. However, ezetimibe has been revealed to provide a by‐product effect on the Nrf2 pathway.^131^ The cardioprotective potential of ezetimibe was evaluated in an animal model of DOX‐induced cardiomyopathy and cardiac cachexia, where ezetimibe attenuated myocardial injury and preserved skeletal and cardiac muscle from atrophy.^132^

Statins, the cornerstone among lipid‐lowering agents (LLAs), are well known for their anti‐inflammatory and antioxidant properties. These agents interact with the Nrf2/ARE system at multiple levels. In an animal model of I/R injury, pravastatin pretreatment reduced oxidative stress and decreased myocardial infarct size.^133^

This effect was mediated by the inhibition of miR‐93 expression, a small non‐coding RNA involved in Nrf2 transcript degradation. Similarly, rosuvastatin demonstrated a cardioprotective effect by increasing Nrf2 and HO‐1 gene expression in both in vitro and in vivo models of TIC.^134^ In this case, the protective mechanism was mediated through activation of the PI3K/Akt signalling pathway.

## What about heart failure with preserved ejection fraction?

**Figure 3:**
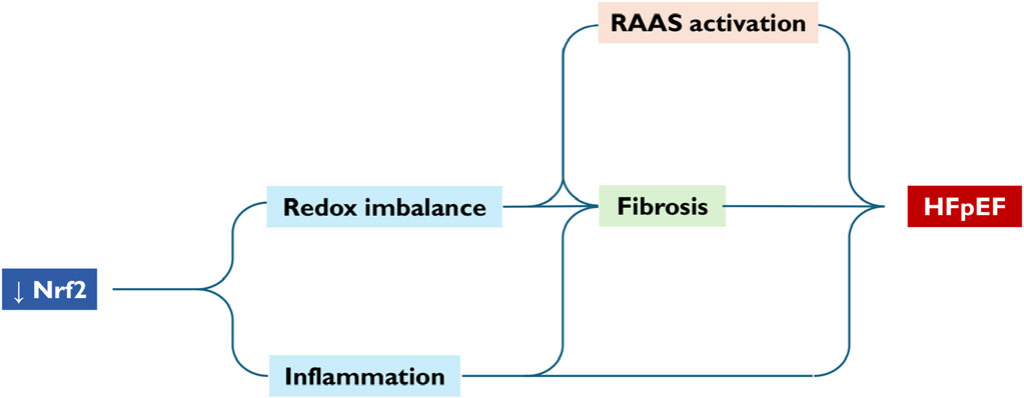
Nrf2 and HFpEF pathogenesis. Reduced Nrf2 activity contributes to redox imbalance and inflammation, triggering fibrosis and activation of the renin–angiotensin–aldosterone system (RAAS). These interconnected pathways promote cardiac hypertrophy and diastolic dysfunction, key features in the development of HFpEF.

Most of the evidence from in vivo studies presented until now are referable to the HFrEF phenotype. In HFpEF, oxidative stress is primarily driven by metabolic and vascular comorbidities such as obesity, diabetes, and hypertension, which induce a sustained pro‐inflammatory environment and endothelial dysfunction.^1^ This milieu promotes microvascular impairment, cardiomyocyte hypertrophy, and interstitial fibrosis, ultimately leading to diastolic dysfunction.^135^ Given this background, HFpEF emerges as a promising target for Nrf2‐based therapy (*Figure* *3*.)

Despite this intriguing hypothesis, few preclinical studies have been conducted in support,^136^, ^137^, ^138^, ^139^ probably also due to the limited availability of reliable animal models that mirror the complexity of HFpEF pathogenesis.^140^ Three out of four studies available investigated the effects of Nrf2 activators in a validated murine model of HFpEF induced by feeding with high‐fat diet (HFD) and N[w]‐nitro‐l‐arginine methyl ester (*L*‐NAME) in drinking water.^136^, ^138^, ^139^

Both melatonin and geniposide (GP), an iridoid glycoside extracted from *Gardenia jasminoides* , were shown to activate Nrf2^136^, ^138^ in obesity‐related HFpEF. The restoration of Nrf2 signalling improved diastolic function, reduced cardiac hypertrophy, and inhibited myocardial ROS production, inflammation, and apoptosis.

Ferroptosis is activated in HFpEF, and the HFpEF hallmarks are functionally and biologically reversible by inhibition of ferroptosis.^141^ Nrf2 activation has the potential to prevent ferroptotic oxidative stress and cell death, and this was demonstrated also in two animal studies on HFpEF.^137^, ^139^ Limonin, a bioactive ingredient derived from citrus fruits, alleviated cardiomyocyte hypertrophy, lipid deposition, and myocardial interstitial fibrosis by modulating ferroptosis‐related pathways.^139^ Limonin was compared with both a control and an empagliflozin treated mouse, mimicking the biological response to the SGLT2i. Finally, another SGLT2i, canagliflozin, showed to regulate ferroptosis, possibly via activating AMPK/PGC‐1α/Nrf2 signalling in HFpEF hypertensive rats.^137^

In conclusion, despite less evidence on the bench, Nrf2 activation appears to confer comparable protective effects in the diastolic phenotype of HF, notably through shared biological pathways such as AMPK activation and ferroptosis inhibition.

## From bench to bedside (and back)

### Experience from clinical trials

Current clinical experience with Nrf2 modulation as a therapeutic target is limited and largely based on evidence from non‐cardiovascular trials.^142^, ^143^ As a result, the impact of these medications in HF patients has been investigated in an exploratory fashion or as part of safety outcomes, making its implications largely speculative or hypothesis‐generating at best.

#### Omaveloxolone

The first example is omaveloxolone, a semi‐synthetic oleanane triterpenoid molecule known for its ability to activate Nrf2.^144^ Omaveloxolone is the only approved medication for Friedreich ataxia (FA), a neurodegenerative syndrome led by mitochondrial dysfunction, energy depletion, and redox imbalance.^145^ The clinical manifestation of this disorder is a cerebellar ataxia; however, the common lethal component in FA is cardiomyopathy and HF. In the MOXIe randomized controlled trial (RCT),^142^ omaveloxolone significantly improved neurological function compared to placebo being generally safe and well tolerated. No imbalances were observed in cardiovascular adverse events such as atrial fibrillation or HF. Treatment with omaveloxolone slightly elevated blood levels of BNP, but this occurred without signs of fluid retention or changes in echocardiograph parameters. This trial, however, excluded patients with reduced LVEF or BNP above 200 pg/mL.^146^ In their study, Salinas *et al*.^147^ demonstrated that omaveloxolone significantly improved contractile function but not survival in a murine model of FA. Neither cardiac fibrosis nor hypertrophy was reversed by omaveloxolone treatment, and impaired mitochondrial function persisted. The authors suggest that either differential biodistribution of the compound between cardiac and neural tissues, or distinct patterns of Nrf2 signalling pathway dysregulation across these organs, may explain the observed neurological benefits, but lack of cardiac improvement, with omaveloxolone.

#### Bardoxolone methyl

Bardoxolone methyl, a synthetic Nrf2 activator analogue of omaveloxolone, has been primarily studied in the context of chronic kidney disease (CKD) and DM.^148^ The phase 3 trial BEACON,^143^ which randomized patients with stage 4 CKD and type II DM to either bardoxolone methyl or placebo, was prematurely terminated due to safety concerns. Specifically bardoxolone treatment provoked a meaningful reduction in urine volume and sodium excretion and an excess of HF events. Two post hoc analysis^149^, ^150^ identified elevated baseline natriuretic peptides and a history of HF hospitalizations as the only factors associated with HF worsening events.^150^

### The ‘dark sides’ of Nrf2

Overall, the selective activation of Nrf2 seems to be detrimental in the clinical context of HF. Preclinical studies identified the triad of reductive stress,^151^, ^152^ misfolded protein toxicity,^153^ and autophagy deficit^154^ as the biological substrate underlying this abnormal response to sole Nrf2 activation in the failing myocyte. Nrf2 largely relies on an efficient autophagic machinery to exert its protective action against mitochondrial dysfunction, oxidative stress, and accumulation of misfolded proteins. At the same time, Nrf2 does not regulate the expression of any autophagy‐related genes, suggesting that Nrf2 may not directly activate autophagy but indirectly facilitates autophagy activation.^155^ In other words, Nrf2 signalling is adaptive in marked abrupt stress when autophagy is exaggerated but is maladaptive during prolonged measured stress when autophagy is suppressed, such is the case of HF.^156^

Sacubitril/valsartan (ARNi) could shed light on the dark side of Nrf2 in the heart.^157^ As demonstrated by our group,^158^ treatment with sacubitril/valsartan increases atrial natriuretic peptide (ANP) levels and restores autophagy and mitophagy in patients with chronic HF. Moreover, the natriuretic effects of ANP and BNP may counteract the fluid and sodium retention observed with Nrf2 stimulation, as seen with bardoxolone methyl.^150^ In this context, sacubitril/valsartan appears to be an ideal companion to an Nrf2 activator in managing oxidative‐metabolic derangement in HF. In the QUEST trial,^159^ the Chinese traditional medicine Qiliqiangxin (QLQX) improved clinical outcomes in patients with HFrEF when added to conventional therapy. QLQX is a commercial formulation containing 11 different plant‐derived ingredients, whose active compounds are primarily AMPK and Nrf2 activators.^156^ Notably, 56.9% of patients in the trial were treated with ARNi. A new clinical trial repurposing an Nrf2 activator (such as bardoxolone methyl) in a contemporary population treated with ARNi could support the hypothesis of an autophagy‐dependent protection.

At present, the most commonly available, widely used, and well‐tolerated Nrf2 activators are the SGLT2i class. SGLT2is are potent activators of Nrf2 via the AMPK/nutrient deprivation pathway, suggesting a potential overlap with the mechanisms of action of QLQX.^156^ Of note, the effect of QLQX on the primary outcome was consistent across all prespecified subgroups, except for the subgroup defined by baseline treatment with SGLT2i. Although several trials have demonstrated their beneficial effects regardless of LVEF phenotype,^160^ a gap in mechanistic evidence on SGLT2i remains. Balanced Nrf2 modulation could largely account for the broad effects of these molecules, yet a study confirming this in humans is still missing.

Another controversy regarding pharmacological manipulation of Nrf2 signalling concerns oncological safety.^161^ Nrf2 relationship with cancer is dynamic and varies depending on the stage of tumourigenesis. Nrf2 is a cellular protector, and this principle applies to both normal and malignant cells.^162^ While Nrf2 activation is cytoprotective in the early stages of transformation,^163^, ^164^its hyperactivation in cancer cells drives malignant progression by enabling uncontrolled proliferation and resistance to apoptosis.^165^ Additionally, the overexpression of ARE‐mediated genes includes multidrug resistance‐associated protein (MRP) efflux pumps, which facilitate chemotherapy resistance.^166^ Sustained activation can shift Nrf2's effect from an initial anti‐inflammatory response towards a state of reduced immunosurveillance, contributing to an environment that favours tumour progression.^167^ Nrf2 reshapes the tumour immune microenvironment (TIME) in favour of angiogenesis and metastasis, mediating the cross‐talk between immunosuppression and cancer promotion.

Despite this strong preclinical foundation, no clinical evidence currently supports a harmful role of Nrf2 activation in tumourigenesis. On the contrary, recent studies and meta‐analyses suggest a favourable oncological safety profile for Nrf2 modulators.^145^, ^168^, ^169^ SGLT2is have demonstrated promising safety and efficacy, even warranting investigation in cancer therapy‐related cardiac dysfunction. Further clinical research is needed to explore the long‐term effects of Nrf2 activation and its potential link to cancer risk.

## Conclusions

HF remains a pathophysiological complex and progressive disease with high morbidity and mortality, demanding novel insights into its progression and therapeutic opportunities. This review underscores the pivotal role of Nrf2 as a master regulator of cellular defence mechanisms, particularly in the context of oxidative stress, mitochondrial dysfunction, and ferroptosis, key drivers in the evolution of HF. In parallel, emerging data illustrate how circulating elements, such as exosomes and leukocytes, interact with the Nrf2 system to propagate systemic oxidative stress and organ cross‐talk, further amplifying myocardial injury.

Beyond its classical antioxidant functions, Nrf2 integrates a complex signalling network that encompasses neurohormonal modulation, chronic inflammation, and IR injury, placing it at the intersection of multiple pathophysiological processes underlying both HFrEF and HFpEF. Translating in the clinical scenario, the dysregulation of Nrf2 contributes to maladaptive remodelling in the context of hypertension, diabetes, and cardiotoxicity, ultimately fostering the transition from compensated cardiac dysfunction to overt HF.

Preclinical evidence consistently highlights the therapeutic potential of pharmacological and natural Nrf2 activators, which mitigate cardiac injury through diverse mechanisms including mitochondrial protection, inflammation resolution, ferroptosis inhibition, and improved metabolic resilience. Moreover, several agents already embedded in guideline‐directed medical therapy (e.g. SGLT2 inhibitors, beta‐blockers, and statins) appear to exert part of their cardioprotective effects via Nrf2 modulation, opening avenues for synergistic treatment strategies.

Despite its therapeutic promise, the clinical translation of pharmacological Nrf2 modulation is still in its early stages. This is partly due to ongoing concerns and controversies regarding potential immunosuppressive and oncological risks. While these safety issues require further clarification through long‐term follow‐up studies, there is already solid evidence supporting the protective effects of SGLT2i, originally developed as antidiabetic agents but, through a process of serendipity, now recognized as modulators of Nrf2.

This emerging paradigm shifts from passive ROS scavenging with exogenous antioxidants to proactive modulation of endogenous cytoprotective pathways. In this context, Nrf2 stands out as an ideal therapeutic target, capable of orchestrating a broad adaptive response that reinforces endogenous resilience, rebalances redox homeostasis, and curtails the pathological cascade driving HF progression.

## Conflict of interest

Emiliano Fiori has been supported by a research grant provided by the DigiCardiopaTh PhD programme.

## Funding

This research did not receive any specific grant from funding agencies in the public, commercial, or not‐for‐profit sectors.
